# COVID-19 as the sole cause of death is uncommon in frail home healthcare individuals: a population-based study

**DOI:** 10.1186/s12877-021-02176-z

**Published:** 2021-04-20

**Authors:** Lena Nilsson, Christer Andersson, Rune Sjödahl

**Affiliations:** 1 Department of Anesthesiology and Intensive Care and Department of Biomedical and Clinical Sciences, Linköping University, S-58185 Linköping, Sweden; 2 Department of Orthopedics, Linköping University Hospital, Linköping, Sweden; 3 Department of Surgery and Department of Clinical and Experimental Medicine, Linköping University, Linköping, Sweden

**Keywords:** Home healthcare, COVID-19, Frailty, Elderly, Mortality

## Abstract

**Background:**

During the first pandemic wave, Sweden experienced a high mortality rate. Home healthcare reflects a group of people especially vulnerable to coronavirus disease 2019 (COVID-19). We aimed to evaluate the pattern of comorbidity and frailty in a group of individuals having fatal outcomes in home healthcare during the COVID-19 pandemic March to September 2020, and to assess the contribution of COVID-19 in the fatal outcomes.

**Methods:**

A cohort of adults with confirmed COVID-19 diagnosis that deceased in home healthcare between March and September 2020 were analysed in a retrospective study comprising home healthcare in 136 facilities in one Swedish county. Main outcome measures were comorbidity and frailty.

**Results:**

One hundred fifty-five individuals (88 women, 67 men) aged 57–106 (median 88) years were included in the analysis. Nine had considerable frailty (ability to perform various activities of daily living but confined to bed or chair on occasion) and the remaining 146 had severe frailty (unable to perform activities of daily living and/or confined to bed or chair; dementia necessitating care). Three or more diagnoses besides COVID-19 were present in 142 individuals and another eight had two diagnoses in addition to COVID-19. In 20 (13%) individuals, COVID-19 was assessed as the principal cause of death, in 100 (64.5%) a contributing cause, and for the remaining 35 (22.5%) death was probably caused by another comorbidity. This seemed to change over the course of the COVID − 19 pandemic, with its contributing role decreasing from the middle of the summer.

**Conclusions:**

Death in home healthcare during the first wave of the pandemic mostly affected individuals with severe frailty and comorbidity at very advanced ages. One fifth of the individuals who died in home health care had another cause than Covid-19.

**Trial registration:**

Clinical Trials.gov NCT04642196 date 24/11/2020.

## Background

Home healthcare mostly targets older people who are frail and have multi-morbidity and thus reflects a group of people especially vulnerable to the coronavirus disease 2019 (COVID-19). It has been in focus due to a disproportionately high number of deaths during the COVID-19 pandemic’s first phase in 2020 [1, 2]. The proportion of individuals in home healthcare who died linked to COVID-19 ranges from less than 0.1% in Singapore and New Zealand to over 5% in six other European countries and the USA, and in Sweden almost half of the deaths in connection with COVID-19 constitutes of individuals in home healthcare [3].

Home-care systems differ between countries [4]. In Sweden, home healthcare is defined as healthcare administered in a patient’s private home or in a special home-care facility and that is consistent over time. Maximal assistance in the patient’s private home including visits 6–8 times a day including night shifts are usually offered before the individual with frailty and multi morbidity is moved to a special home-care facility. Among Swedish individuals at age 80 years or more, 13% of women and 20% of men live in a home-care facility [5]. These facilities are regarded as the individual’s home. Family members can visit freely. The care provided including diagnostic possibilities are equivalent to what can be offered in an ordinary home. Registered nurses provide the highest medical competence in home healthcare. Physician resources—regular, telephone, or on-demand consultations—are provided by primary care physicians.

For the elderly with multi-morbidity, reports have suggested that the clinical manifestations of COVID-19 may be difficult to recognise because typical symptoms such as fever, cough and dyspnoea may already be present due to other comorbidities like several pulmonary or cardiac diseases [6]. COVID-19 may also have non-specific and/or atypical presentations like anorexia, diarrhoea, fatigue, headache and dizziness [7–9]. Elderly patients with COVID-19 seem to decline suddenly [10]. Accordingly, advance care planning is crucial, including plans for palliative care in the home-care setting if it is determined that hospital care would be of no benefit for the individual.

In a cohort of individuals cared for in home healthcare with positive detection of SARS-CoV-2 nucleic acid for the COVID-19 diagnoses and a fatal outcome during the first pandemic wave, we aimed to evaluate comorbidity and frailty, and secondly to assess the contribution of COVID-19 to those fatal outcomes.

## Methods

### Study design and oversight

We undertook a retrospective descriptive cohort study in Östergötland County (465,500 inhabitants), Sweden, including all individuals cared for in home healthcare who died between March and September 2020 and had a laboratory confirmed positive COVID-19 diagnosis. Individuals that were transferred to hospital care were excluded. Home healthcare in the county included 136 home healthcare facilities and also individuals cared for in their own private homes.

The Swedish Ethical Review Authority, Göteborg avdelning 2 medicin; chairman Bengt Nilsson, approved the study (Dnr 2020–03029 and 2020–04443), undertaken in accordance with the Declaration of Helsinki. The ethics authority has waived the requirement of informed consent for this study as the structured record review was a part of mandatory patient safety work demanded by Swedish legislation. The authors designed the study, collected the data, and performed the analyses.

### Data collection

The medical records of all individuals in the cohort were retrospectively reviewed by two senior experts in patient safety and record review (RS, CA). The COVID-19 diagnosis was based on the detection of SARS-CoV-2 nucleic acid using PCR technique or, less frequently, by virus isolation. Clinical or radiological signs of COVID-19 alone did not qualify for inclusion in the study. The review focused on the period from the start of the COVID-19 disease to the individual’s death.

From the records, we collected patient-level data on demographic variables, clinical signs, comorbidity, performance status, and cause of death. Further, we ascertained whether a written plan for care in case of new symptoms or impairment was at hand before the diagnosis of the COVID-19 disease.

### Definitions and outcomes

Comorbidity beside the COVID-19 diagnosis was graded into three groups: 0–1, 2, and 3 or more diagnoses.

The combined assessment of performance status and frailty was adapted to the record review method and describe the individual’s status during the month preceding the COVID-19 disease. We used a modification of the WHO/ECOG performance status [11] and frailty score according to Rockwood [12] and classified them into four groups:

- None or mild frailty: no restrictions in daily life.
- Moderate frailty: mobile and independent but unable to handle physically demanding activities or work.
- Considerable frailty: able to perform activities of daily living but confined at times to bed or chair.
- Severe frailty: unable to perform activities of daily living and/or confined to bed or chair. Dementia necessitating care.

In the assessment of cause of death, we identified three groups: COVID-19 was the fully dominating cause; COVID-19 contributed in conjunction with other comorbidities; death was most probably caused from a disease other than COVID-19. In the process of assessing the cause of death, we evaluated the time between the confirmed diagnosis and death. In this decision, the individual’s age was not taken into account.

### Statistical analysis

Descriptive statistical analysis was undertaken and presented as median (range) or n (%).

## Results

One hundred and fifty-five individuals, 88 women (57%) and 67 men (43%) aged 57–106 (median 88) years, were included in the analysis. All subjects had considerable or severe frailty, and the vast majority had ≥3 diagnoses besides COVID-19 (Table 1).

**Table 1:** Frailty and comorbidity among 155 deceased individuals in home healthcare diagnosed with COVID-19

	N (%)
Frailty:	
None/mild frailty	–
Moderately frail	–
Considerably frail	9 (6%)
Severely frail	146 (94%)
Comorbidity*:	
0–1	5 (3%)
2	8 (5%)
≥ 3	142 (92%)

*Number of diagnoses besides COVID-19

**Fig. 1:**
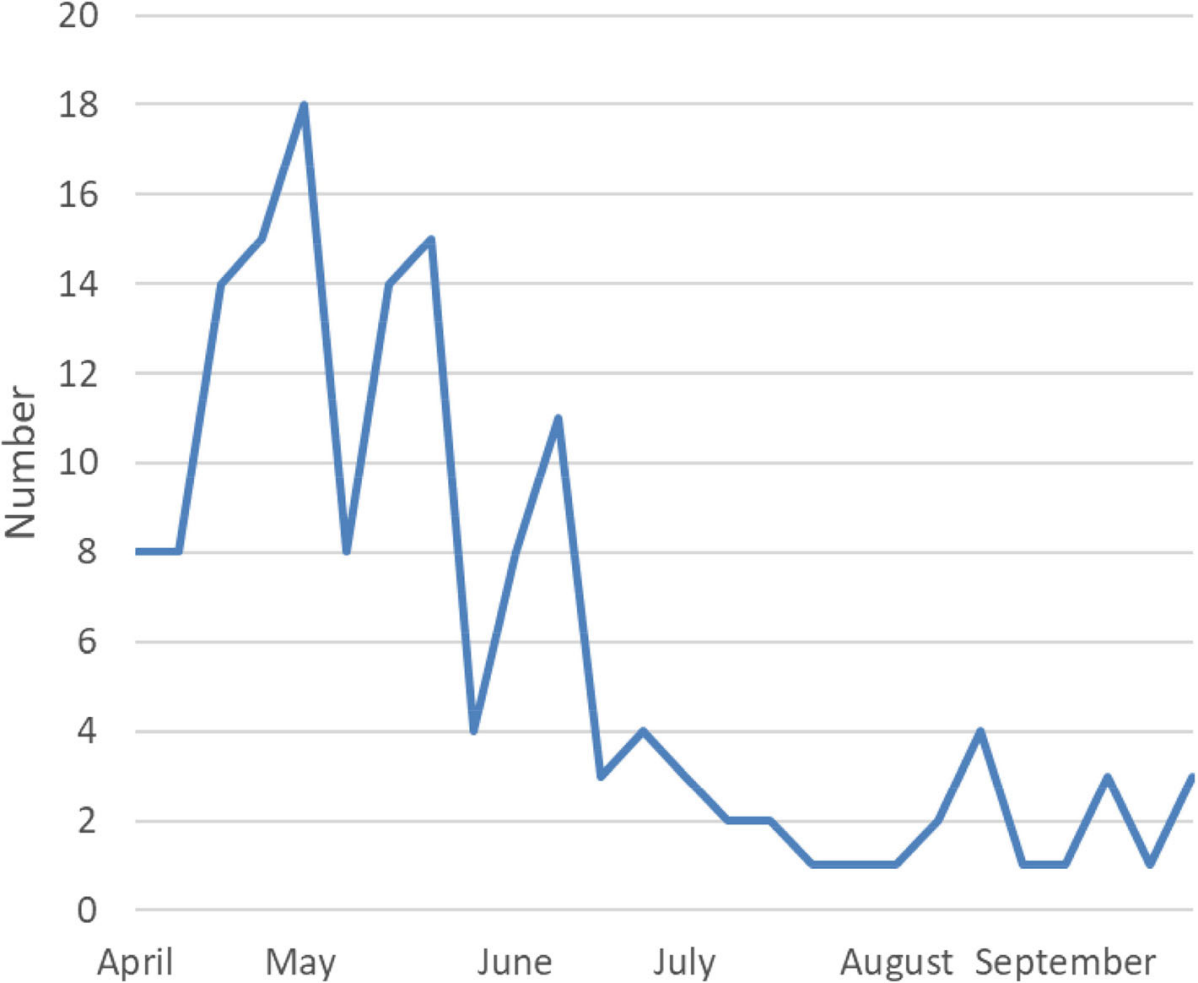
Number of deaths per week from April to September 2020

The first death occurred during Week 14 (starting March 30). From the middle of June, the death rate was less than five per week (Fig. 1).

**Fig. 2:**
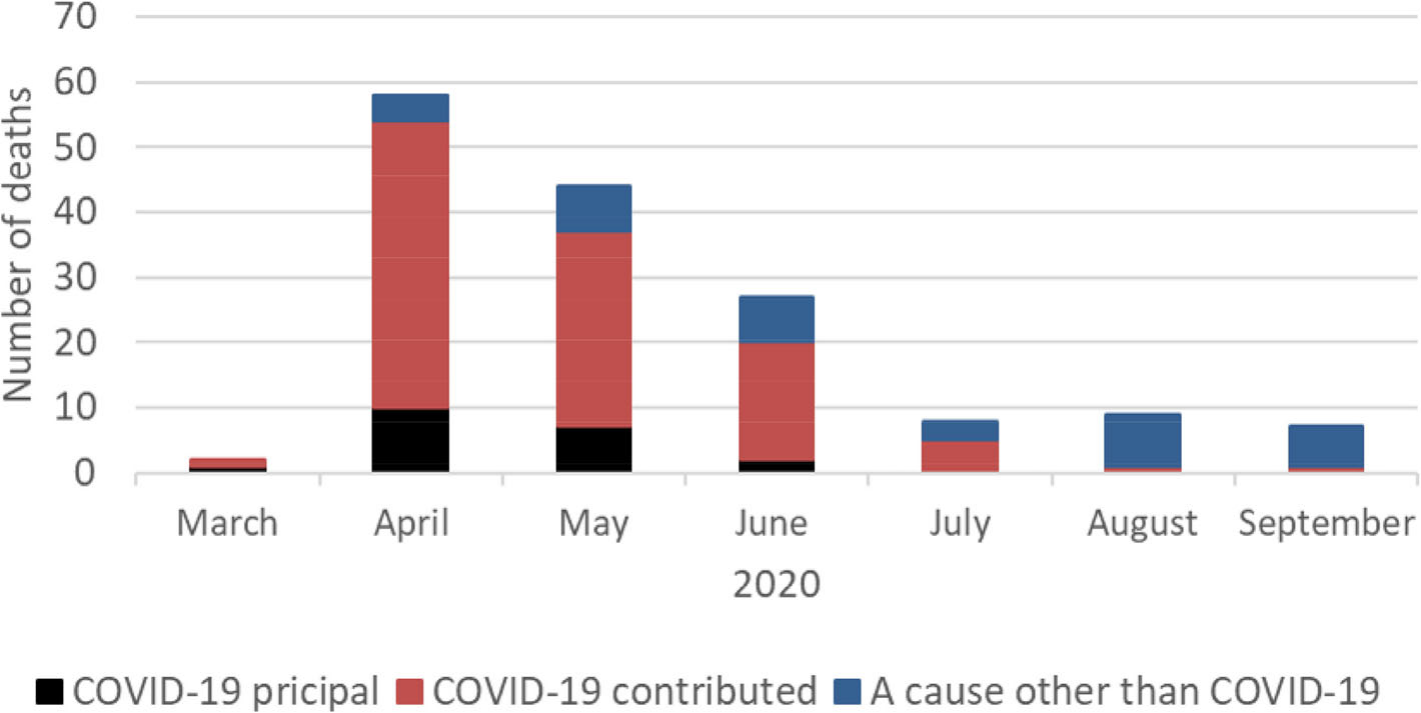
Assessed principal cause of death over time

In 20 individuals (13%), COVID-19 was assessed as the principal cause of death, in 100 (64.5%) as a contributing cause, and in the remaining 35 (22.5%), death was probably caused by another comorbidity. This changed over time (Fig. 2).

**Fig. 3:**
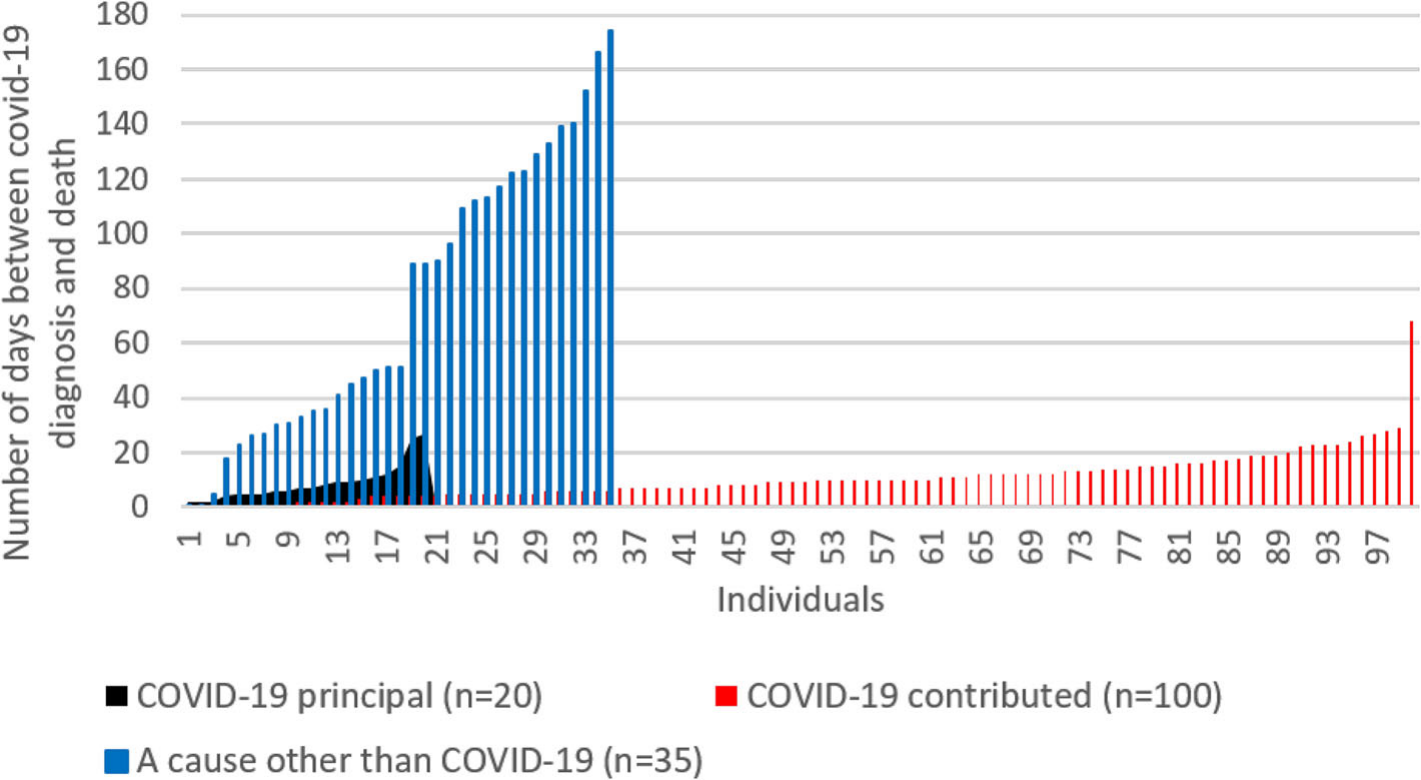
Time between positive COVID-19 diagnosis and death divided by assessed cause of death. Each bar on the X-axis represents one individual, and the three groups overlap. Thirty days or more after a confirmed diagnosis of COVID-19, the disease was assessed as a contributing cause of death in only one person and in none as the principal cause

The time between the positive PCR test for SARS-CoV-2 nucleic acid and death was a median of 10 (1–174) days. The longer the time between a positive COVID-19 diagnosis and death, the lower the probability of a causative correlation (Fig. 3). Thirty days or more after a confirmed diagnosis of COVID-19, the disease was assessed as a contributing cause of death in only one person and in none as the principal cause.

Typical clinical signs in the early phase of COVID-19 were dyspnoea, cough, high respiratory rate, desaturation, diarrhoea, fatigue, chills, and fever. We also noticed thrombosis, impaired smell, taste, and other affected cerebral functions, as well as impaired renal function. Later on, during the last days of life, upper respiratory symptoms were not prominent. Instead, general deterioration and fatigue were more common.

A plan for care in case of deterioration was documented in 139 (90%) of the individuals’ records. In 16 (10%), no plan was found in the medical record. Often, an active decision about changing from active care to terminal care was documented. Accordingly, fluid resuscitation and oxygen therapy were rarely used. Morphine, haloperidol, and midazolam often constituted the base for symptom relief in a terminal phase.

In total, 43 (32%) of 136 home-care facilities were affected by deaths among residents with a positive COVID-19 diagnosis. In eight of them, five or more patients died. Thirty individuals died in their private homes.

## Discussion

### Principal findings

Death in home healthcare during the first pandemic wave mostly affected individuals already vulnerable due to severe frailty and very advanced age. In this group of subjects, COVID-19 was assessed as contributing to death in two-thirds of the individuals, and less frequently, it was the dominant cause of death (13%). One of every five individuals was assessed as dying from another cause than COVID-19.

### Strengths and limitations

A principal strength of our study is that it is population-based, as we included all home healthcare facilities during the first pandemic wave in Östergötland county with almost half a million inhabitants. Other strengths are that two reviewers are experts in the field of record review and patient safety who made careful reviews, and the laboratory tests used for the COVID-19 diagnosis that constituted criteria for inclusion in the study were highly reliable. A limitation is that the data were collected retrospectively and only symptoms and findings documented in the medical records could be evaluated. The documentation in home healthcare was unfortunately more limited than that during hospital stay. In the beginning of the study, the capacity of testing for COVID-19 was limited and only offered to individuals with symptoms. During the first wave, the knowledge that discrete symptoms in this group of individuals, i.e. anorexia and general deterioration, could be the only signs of COVID-19 grew. Early during the first wave it is probable that our inclusion did not cover all individuals as testing took place in connection with more “classical” respiratory symptoms and/or fever.

An incentive of the study was to try to determine to what extent COVID-19 per se affected mortality rates. For individuals who developed characteristic respiratory symptoms and fever leading to death within a short period of time (a few weeks) COVID-19 was judged as the dominant cause of death. On the other hand, for individuals without typical symptoms for COVID-19 but with another life-threatening disease (i.e. advanced cancer) and when there was a delay between confirmed diagnosis of COVID-19 and death, the cause was not judged to be COVID-19. However, in two thirds of individuals who died in home health care, the cause of death was less obvious. Symptoms of COVID-19 were vague and there were other possible causes for death like ischemic cardiac disease, chronic pulmonary disease or dementia. For such individuals COVID-19 was judged as a contributing cause of death. This study demonstrates that comparison of mortality rates after COVID-19 in home health care should be interpreted with caution.

The term frailty is used to describe loss of reserves in multiple dimensions—energy, physical ability, and cognition—and health conditions that give rise to vulnerability. There are several established scoring systems designed to describe function loss due to frailty, and they must be collected in direct personal contact [12]. As our study was a retrospective record review, we found it necessary to modify and simplify the scoring. We therefore decided to categorize frailty in four groups. The impact of comorbidity on mortality may vary by the degree to which the various diseases are compensated. A well-controlled disease will probably not have the same risk for high mortality as a disease not being adequately controlled. The degree of comorbidity is therefore associated more closely with frailty than with the number of diagnoses.

In a Swedish single-centre observational study on patients admitted to geriatric hospital care between March and June 2020, in-hospital mortality was 24% [13]. Age, frailty in terms of a Clinical Frailty Scale over 5, and Charlson Comorbidity Index were predictive for in-hospital mortality. In a Spanish study predictors of mortality for home healthcare residents were being male, older than 80 years, admitted to a hospital for COVID-19, and having cardiovascular disease, kidney disease or dementia [14]. This is in line with our study, in which severe frailty and several comorbidities were present in diseased individuals. About 36% of all deaths in Sweden 2018 took place in home healthcare [15] In the largest Swedish town area, the proportion of patients dying in nursing homes as a fraction of all deaths in 2020 was 32% in March, 43% in April, and 34% in May [16].

A frail immune system is characterised by alterations in T-lymphocyte production and blunting of the B-cell-led antibody response. The immune system thus fails to respond to acute inflammation. An abnormal low-grade inflammatory response plays an important role in the pathophysiology of frailty [12, 17]. Poor nutritional status in the frail individual also contributes to poor immunity [18].

Estimation of the death toll from natural disasters, including pandemics, is challenging. Two approaches are commonly used. The first is estimation of extra deaths relative to the same period in preceding years, which can be limited by wide confidence intervals [19]. The second is the body-count approach with thorough analysis of records and death certificates. The latter is limited by the quality of documentation and interpretation of data. In a study from nursing homes in London, the medical certificate listed confirmed or suspected COVID-19 as the underlying cause of death in just over half of the residents [20]. The deaths coded as non-COVID happened earlier in the outbreak than the COVID-19-associated deaths, and included frailty or old age interacting with other comorbidities, like dementia. In the present study, if a long time (over 30 days) had elapsed and clinical signs of an ongoing infection were absent, we found it more probable that another cause than COVID-19 had caused the death. Instead, a principal cause in our study was heart failure.

A point-prevalence study in UK nursing homes showed that besides typical symptoms like fever and cough, many residents developed atypical or no discernible symptoms. New-onset anorexia showed the strongest association with SARS-CoV-2 positivity [20]. Thus, it has been suggested that COVID-19 should be suspected in any resident with airway symptoms or fever, as well as loss of appetite and reduced oral intake, new-onset/worsening confusion, or any subtle sign of deterioration. This knowledge was not present in the first phase of the pandemic. Accordingly, home healthcare residents with subtle signs were probably not tested in our county during our first studied months and were not included in our studied cohort.

It must be carefully considered if or in what situations hospital admission is indicated for elderly and frail individuals receiving home healthcare. In our cohort, 90% had documented advance care plans. All treatments can cause harm as well as benefits. Advance care planning, including plans about escalation to hospital, is essential. Discussions should include residents and their families and cover COVID-19 as well as comorbidities per se. According to British guidelines, specific interventions connected to COVID-19 (i.e. oxygen and subcutaneous fluids) are often inappropriate; treatments focusing on symptom control may be better when a resident is nearing the end of life [21]. In our cohort, morphine, haloperidol, and sedatives were used for both palliation and terminal care. We observed that the possibility of administering oxygen and parenteral fluid was not frequent. Another reflection is that increased participation of physicians would be desirable. From a systematic retrospective record review of all hospitalised patients with COVID-19 in our county, we noticed around 40 patients from the home healthcare setting were receiving care with a mortality of around 40% (unpublished results).

Preventing the spread of SARS-CoV-2 into the care home setting is particularly important in preventing outbreaks. During the first pandemic wave in 2020, family members and friends were prohibited from visiting care homes according to rules from the Public Health Agency of Sweden. This did not prevent the infection from reaching the home-care facilities. High turnover of staff and a large number of substitutes are problems in Swedish home healthcare. This challenges the implementation of good preventive care hygiene rules. In New York State, the presence of a healthcare workers’ union was associated with a 30% lower mortality rate from COVID-19 among nursing home residents and greater access to personal protection equipment [22].

## Conclusions

We found that death in home healthcare in a cohort with positive detection of SARS-CoV-2 nucleic acid for the COVID-19 diagnoses during the pandemic’s first wave in 2020 mostly affected individuals with severe frailty and very advanced age. Therefore, COVID-19 was regarded as having no obvious role in the death of about 20% of the subjects. Comparison of mortality rates after COVID-19 in home health care should be interpreted with caution.

## Data Availability

The datasets generated and/or analysed during the current study are not publicly available due to the integrity of patients records, but the summarised findings are available from the corresponding author on reasonable request.

## Abbreviation

COVID-19: Coronavirus disease 2019
